# p300- and Myc-mediated regulation of glioblastoma multiforme cell differentiation

**DOI:** 10.18632/oncotarget.139

**Published:** 2010-08-04

**Authors:** Sreejith P. Panicker, Baisakhi Raychaudhuri, Pankaj Sharma, Russell Tipps, Tapati Mazumdar, Asoke K. Mal, Juan M. Palomo, Michael A. Vogelbaum, S. Jaharul Haque

**Affiliations:** ^1^Department of Cancer Biology, Lerner Research Institute, Cleveland Clinic, 9500 Euclid Avenue, Cleveland, Ohio 44195, USA; ^2^Brain Tumor & Neuro-Oncology Center, Cleveland Clinic, 9500 Euclid Avenue, Cleveland, Ohio 44195, USA; ^3^Department of Cell Stress Biology, Roswell Park Cancer Institute, BLSC 3319 Elm and Carlton Streets, Buffalo, New York 14263, USA; ^4^Department of Neurosurgery, Cleveland Clinic, 9500 Euclid Avenue, Cleveland, Ohio 44195, USA; ^5^Department of Pulmonary, Allergy and Critical Care Medicine, Cleveland Clinic, 9500 Euclid Avenue, Cleveland, Ohio 44195, USA

**Keywords:** glioblastoma multiforme (GBM), GBM stem cell, differentiation, invasion, p300, Myc

## Abstract

Tumorigenic potential of glioblastoma multiforme (GBM) cells is, in part, attributable to their undifferentiated (neural stem cell-like) phenotype. Astrocytic differentiation of GBM cells is associated with transcriptional induction of Glial Fibrillary Acidic Protein (GFAP) and repression of Nestin, whereas the reciprocal transcription program operates in undifferentiated GBM cells. The molecular mechanisms underlying the regulation of these transcription programs remain elusive. Here, we show that the transcriptional co-activator p300 was expressed in GBM tumors and cell lines and acted as an activator of the GFAP gene and a repressor of the Nestin gene. On the other hand, Myc (formerly known as c-Myc overrode these p300 functions by repressing the GFAP gene and inducing the Nestin gene in GBM cells. Moreover, RNAi-mediated inhibition of p300 expression significantly enhanced the invasion potential of GBM cells in vitro. Taken together, these data suggest that dedifferentiated/undifferentiated GBM cells are more invasive than differentiated GBM cells. Because invasion is a major cause of GBM morbidity, differentiation therapy may improve the clinical outcome.

## INTRODUCTION

Glioblastoma multiforme (GBM), the highest (IV) grade of malignant gliomas, is the most common tumor of the central nervous system [1, 2]. The mean survival of GBM patients is about one year despite use of surgery, radiotherapy, and chemotherapy [1, 2]. Development of effective therapies for GBM must require a better understanding of the biology of cells that cause and drive the disease [1, 3]. Recent studies suggest that undifferentiated, stem cell-like neoplastic cells, known as ‘cancer stem cells’ (CSCs), but not differentiated GBM cells, drive cancer maintenance in rodent xenograft models [4, 5, 6, 7]. In GBM, CSCs termed ‘GBM stem cells’ (GSCs) are highly proliferative, angiogenic and resistant to radiotherapy and chemotherapy [1, 2, 4, 7, 8].

Invasion is a defining hallmark of GBM [2, 9, 10, 11]. Intriguingly, striking resemblances are found between the migratory properties of neural stem cells (NSCs) and invasive GBM cells [1, 12, 13]. Invasion of GBM cells that are resistant to radiotherapy and chemotherapy largely contributes to the recurrence of the tumor [9, 14]. Therefore, the use of differentiation therapy, in combination with the conventional modalities, including surgery, radiotherapy and chemotherapy, may improve the clinical outcome. However, the molecular mechanisms underlying the astrocytic differentiation of GSCs remain unclear. Differentiation of normal NSCs is associated with a reprogramming of the expression of the intermediate filamentous proteins, including Nestin and Glial Fibrillary Acidic Protein (GFAP). Nestin is expressed in both NSCs and GSCs, and astrocytic differentiation of both cell types is associated with repression of the Nestin gene and concomitant induction of the GFAP gene [15, 16, 17, 18].

The transcriptional co-activator p300, which is expressed in GBM cells, acts as a key regulator of transcription in a context-dependent fashion by interacting with a variety of proteins, including Stat3, Smad1/4, and Notch1, which play distinct roles in astrocytic differentiation [19, 20, 21]. p300 also acts as an activator of muscle differentiation [20]. Myc, a nuclear oncoprotein formerly known as c-Myc, forms dimer with Max, which recognizes the E-box sequence located in the regulatory regions of a variety of genes that regulate cell proliferation, differentiation and apoptosis [22, 23]. Myc is expressed in GBM cells [23, 24, 25]. Although amplification, rearrangement and overexpression of the Myc are rarely found in malignant gliomas [26, 27], the half life of Myc protein remains 4-6-fold elevated in a number of glioma cell lines, suggesting that Myc stabilization may be linked to the pathogenesis of GBM [28]. A recent report reveals that simultaneous inactivation of p53 and PTEN promotes an undifferentiated phenotype of mouse NSCs, which is associated with increased expression of Myc [29]. Further, Lassman *et al.* have reported that overexpression of Myc represses GFAP expression with a concomitant activation of the Nestin gene in mature murine astrocytes, making them morphologically similar to NSCs [30]. Another study shows that Myc is required for the proliferation and survival of GSCs [31]. Interestingly, p300 plays dual roles in Myc regulation: as a co-activator of Myc by stabilizing Myc protein and as an inducer of Myc instability by directly acetylating Myc [32].

Here, we show that in GBM cells, p300 acted as an activator of the GFAP gene and a repressor of the Nestin gene, whereas Myc opposed these p300 functions. Moreover, the tumorigenic potential of GBM cells was reciprocally associated with their astrocytic differentiation and p300 markedly suppressed the invasion capacity of GBM cells *in vitro*.

## MATERIALS AND METHODS

### Cell culture and reagents

GBM cell lines U87, U251, SNB19, D54 and LN229 and human embryonic kidney cell line 293T were cultured in DMEM supplemented with 10% heat-inactivated fetal bovine serum (Serum Source International Inc, Charlotte, NC, USA), 2 mM L-glutamine and 50 mg/l of penicillin G and streptomycin. Lipofectamines and Alexafluors were purchased from Invitrogen (Carlabad, CA, USA). GFAP antibody, MTT assay reagents and Luciferase assay kit were purchased from Promega (Madison, WI, USA) and site-directed mutagenesis kit was from Stratagene (La Jolla, CA, USA). Nestin antibody, p300 antibody and EZ-ChIP reagents were purchased from Millipore (Temecula, CA, USA). shRNAs for p300 and Myc were from Origene Tech. Inc. (Rockville, MD, USA). IL-6 and EGF were obtained from R & D Systems (Minneapolis, MN, USA). Myc antibody and β-actin antibody were purchased from Santa Cruz Biotech. Inc. (Santa Cruz, CA, USA). Matrigel invasion chambers were obtained BD Biosciences (San Jose, CA, USA).

### Transient and stable transfection of GBM cells

U87, D54, SNB19, and LN229 cells were transfected with plasmid DNA using lipofectamine 2000 and U251 cells using Lipofectamine Plus. 293T cells were transfected using calcium phosphate and DNA co-precipitation method as described previously [33]. For generation of stable clones, U251 cells expressing p300-shRNA, cells were selected for 2-3 weeks in the presence of 0.5 µg/ml of puromycin [34].

### Tumor implantation

Implantation of the GBM cells in immune compromised rats and mice was performed in accordance with protocols approved by the Cleveland Clinic Institutional Animal Care and Use Committee, as described [34]. Briefly, four weeks old athymic male rats (Charles River, National Cancer Institute) were anesthetized using ketamine hydrochloride (60 mg/kg) and xylazine (5 mg/kg). Head was carefully cleaned with iodine, and a fresh suspension of 5×10^5^ GBM cells in 5 µl PBS was injected stereotactically in the right frontal lobe through a burrhole. After 3 weeks, volumes of intracranial tumors were measured by pixel imaging analysis. For subcutaneous tumors, 2.5×10^6^ GBM cells were mixed with matrigel (1:5) and injected in right flanks of 4 week old male nude athymic mice (Charles River, National Cancer Institute). Volumes of tumors were measured after 5 weeks using the formula: volume = width^2^ × length × 0.4 [35]. Five animals were used for each GBM cell line.

### Luciferase reporter constructs and Luciferase assay

The GFAP-luciferase reporter (provided by Dr. Abhijit Guha, The Hospital for Sick Children, Toronto, Canada) is driven by a 2.21 kb human GFAP promoter [36,37]. A 714 bp NSC-specific Nestin enhancer (located in the second intron of the human Nestin gene) [18,38,39] was PCR-amplified using a template of genomic DNA isolated from U87 cells. The amplified Nestin enhancer fragment was cloned into the Bgl II restriction site of TP222 vector in which the luciferase gene was driven by an 81 bp minimal thymidine kinase (TK) promoter [40]. The GAS enhancer TTCCGAGAA mutated to TGCCGAGTA in the human GFAP promoter by site-directed mutagenesis as described [34,41]. The luciferase activity was measured and normalized as described earlier [42]. Cells were cotransfected with Renilla luciferase construct (driven by TK promoter) and GFAP-luciferase or Nestin luciferase. Normalized values are presented in terms of percentage luciferase activity for comparison between the two cell lines U87 and U251 used, and among the different experimental conditions employed.

### Immunofluorescence and immunohistochemistry

For detection of GFAP and Nestin expression by immunofluorescence, cells were grown on coverslip, fixed (30 min) with 4% paraformaldehyde followed by permeabilization (10 min) with 2% paraformaldehyde and 0.2% Triton X-100 and blocked (1 h) in PBS containing 3% goat serum and 0.3% Triton X-100. Cells were incubated with GFAP (1:1000) or Nestin (1:200) antibody overnight at 4°C. Alexaflour488- and Alexaflour568-conjugated secondary antibodies were used for the detection of GFAP and Nestin respectively. For detection of p300 and Myc in GBM tumors by immunohistochemistry, 8 µm sections of frozen tumor samples were fixed with 4% formaldehyde and permeabilized with 0.3% Triton X-100. Endogenous peroxidase activity was quenched with 0.3% H2O2 in PBS containing 0.3% normal goat serum (NGS) and blocked using 10% NGS. Samples stained with either anti-p300 (1:200) or anti-Myc (9E10, 1:100) followed by biotinylated goat-anti-rabbit (1:500) for p300 or biotinylated goat-anti-mouse (1:200) for Myc. Samples were then treated using the Vectastain Elite ABC Kit (Vector Labs) and visualized using DAB Substrate Kit (Vector Labs). Samples were counterstained with methyl green (0.5% methyl green; 0.1M sodium acetate) at 60°C for 5 min, dehydrated and mounted using Vecta-Mount (Vector Labs). Appropriate negative controls were included for all samples. Images were collected at 20X magnification.

### Chromatin immunoprecipitation

For p300 ChIP, cells were grown in serum free medium for 4 h followed by the appropriate cytokine treatment. U251 and U87 cells were treated with IL-6 (20 ng/ml) or EGF (100 ng/ml), respectively, for 30 min. For Myc ChIP, cells were grown in serum free medium for 48 h followed by subsequent incubation with 20% serum containing medium for 4 h. Following appropriate treatments, U251 and U87 cells were treated with 1.5 mM ethylene glycol bis[succinimidylsuccinate] (EGS, Thermo Scientific) for 20 min followed by 1% formaldehyde for 10 min [43,44]. Cross-linking was subsequently quenched by treatment with 125 mM glycine for 5 min. ChIP assays were carried out using 2.0 µg of p300-or Myc antibody following the EZ-ChIP protocol (Millipore). DNA was purified using the UltraClean PCR Clean-up Kit (Mobio; Carlsbad, CA, USA) following the manufacture’s instructions. PCR was carried out using ^32^P-labeled primers for the GFAP promoter to amplify the −2139 to −1824 E-Box containing region [(5’-TGCTGGGACTCACAGAGGGAGACC-3’ (Forward) and 5’TGGCGCAACCACGACTCACT (Reverse)] and the Nestin enhancer −414 to −166 region [(5’-CTCCTTCTCAGACCCTCCAG-3’ (Forward) and 5’TCACATACCCACAGACATCACA-3’ (Reverse)] using optimized reaction conditions. PCR products were resolved by 6% polyacrylamide gel eletrophoresis and visualized using a Storage Phosphor Screen and scanned using the Storm 840 Imager (Molecular Dynamics). Band densities were determined using ImageQuant 5.2 (Molecular Dynamics) and calculated to represent ‘percent of input’.

### Cell invasion, migration and proliferation

The invasion potential of U251 cell lines was measured using modified Boyden chamber containing matrigel (BD Biosciences) [45,46,47]. Membrane containing 8 µm pores in each well was coated with a basement membrane matrix (matrigel). 2.5 × 10^4^ U251 cells stably expressing p300-shRNA (Sh #1-clone 10 or Sh #3-clone 10) in 500 µl media containing no growth factors were seeded on top of the matrigel. The bottom well contained media with growth factors. Cells were incubated for 24 and 48 h. Cells that remained on the top of the membrane were scrubbed off with cotton swabs. Cells invaded into the matrix were fixed, stained with hematoxylin blue and counted using the ImageQuant software as described [46]. Cell migration was determined *via* wound healing assays as described [48]. Briefly, cells were seeded in 6-well plates and grown to 100% confluent monolayer, and scratched by a pipette-tip, washed with PBS and incubated in serum-free medium for 24 or 48 h. Then cells were stained with Gemisa stain and phase contrast images were taken. Cell proliferation was determined using MTT assay by absorbance at 490 nm employing a 96-well plate reader as described [49].

### Western blot analyses

For detection of protein levels, whole cell lysates were prepared by lysing cells in ice-cold buffer composed of 50 mM Tris-HCl (pH 7.9), 150 mM NaCl, 1 mM EDTA, 1 mM DTT, 1% NP-40, 10% glycerol, 1 mM PMSF, 2 µg/ml leupetin, 2 µg/ml pepstatin, and 5 µg/ml aprotinin, on ice for 30 min. SDS-PAGE and Western blot analyses were performed using standard procedures [41,42].

### Statistical Analyses

Results are presented as Mean ± SE from at least three independent experiments. Prism software was used to perform Student’s *t-*test and differences between values will be considered significant when P < 0.05.

## RESULTS

### Tumorigenic potential of GBM cells correlates with GFAP and Nestin expression

**Figure 1:**
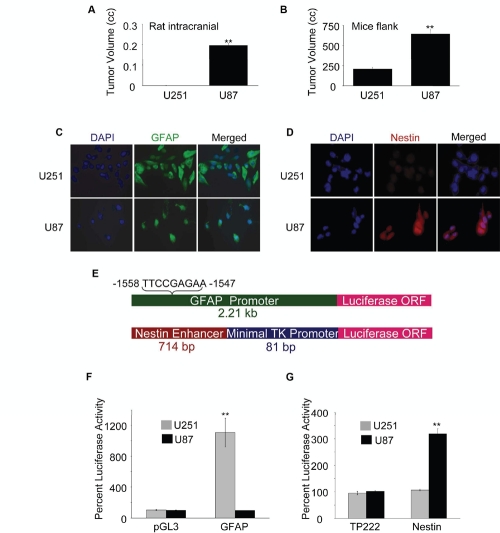
Tumorigenicity of GBM cells correlates with expression of Nestin and GFAP (A) Rat right frontal lobes were implanted with 5×10^5^ U251 or U87 cells in 5 µl PBS. After 3 weeks, tumor volumes were determined by pixel imaging analysis. (B) The right flanks of nude mice were injected (s.c.) with 2.5×10^6^ U251 or U87 cells in 100 µl PBS (mixed with 100 µl matrigel). After 5 weeks, tumor volumes were calculated using the formula: volume = width^2^ × length × 0.4. U251 or U87 cells grown on coverslips were fixed, permeabilized and stained for (C) GFAP (green) and (D) Nestin (red) using respective primary antibodies and secondary antibodies conjugated with Alexafluor488 and Alexafluor568 respectively. (E) Schematic of luciferase (Luc) reporter constructs: GFAP-Luc constitutes a 2.21 kb human GFAP promoter containing a GAS element (−1558 to −1547) in pGL3-Basic vector (upper panel) and Nestin-Luc constitutes a 714 bp enhancer fragment form the second intron of the human Nestin gene cloned upstream of an 81 bp minimal thymidine kinase (TK) promoter driving the luciferase gene in the vector, TP222 (lower panel). Activities of the GFAP promoter (F) and the Nestin enhancer (G) were determined at 72 h post transfection of U251 and U87 cells (1×10^6^) with indicated reporter plasmids (or empty vectors) by luciferase assay. For (A) & (B), each value represents mean ± SE of 5 individual animals of each group. Normalized percent luciferase values for (F & G) are plotted as mean ± SE (n = 3). ** indicates p < 0.01

It is well documented that GFAP expression correlates with the astrocytic differentiation of GBM cells, while a high level of Nestin expression is detected in undifferentiated neural precursor cells [1,2,4,15]. We have investigated the tumor forming potential of two GBM cell lines, U251 and U87, expressing different levels of GFAP and Nestin. We found that U87 cells formed significantly larger tumors than U251 cells in immune compromised rodents when implanted in frontal brain lobes of rats (Fig. 1A), and right flanks of mice (Fig. 1B). We found differential expression of GFAP and Nestin in these two cell lines: while GFAP levels were much higher in U251 cells compared with U87 cells (Fig. 1C), Nestin expression pattern was in reverse to that of GFAP with high levels in U87 cells and almost undetectable in U251 cells (Fig. 1D).

To determine the transcriptional activity of GFAP and Nestin in U251 and U87 cells, we used luciferase reporter constructs driven by either a 2.2 kb human GFAP promoter [36,37] or a 714 bp NSC-specific Nestin enhancer (located in the second intron of the human Nestin gene) [18,38,39] linked to a minimal thymidine kinase (TK) promoter [40] (Fig. 1E). We found that U251 cells had high transcriptional activity for GFAP but undetectable Nestin transcription. In contrast, U87 cells exhibited undetectable GFAP transcription and high Nestin enhancer activation (Figs. 1F and G). Taken together, these results suggest that the tumorigenic potential of GBM cells is reciprocally associated with the level of differentiation. These observations are in agreement with earlier reports for the tumorigenic potential of U251 cells and their expression levels of GFAP and Nestin [4,12,50,51,52,53].

### p300 differentially regulates the expression of GFAP and Nestin in GBM cells

**Figure 2:**
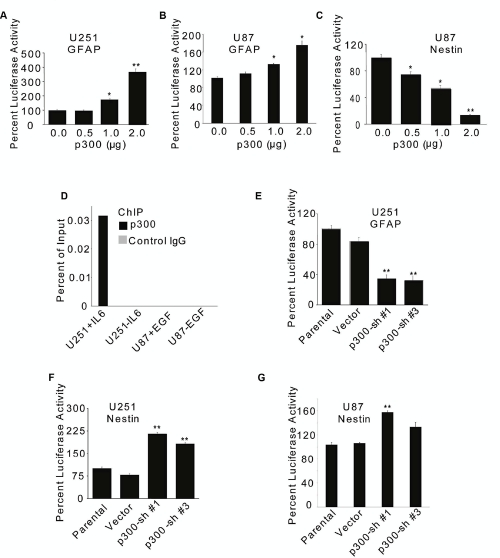
p300 differentially regulates transcription of GFAP and Nestin genes U251 (A) and U87 (B & C) cells (1×10^6^) were transfected with 2 µg GFAP-Luc (A & B) or 2 µg Nestin-Luc (C) reporter constructs along with indicated amounts of p300 or empty control vector (A, B & C). Luciferase activity was measured at 72 h after transfection. (D) 2×10^6^ cells treated with 20 ng/ml of IL-6 or 100 ng/ml of EGF for 30 min and subjected to ChIP using anti-p300 or matched IgG antibodies. p300 occupancy to the GFAP promoter was determined by PCR using radio-labeled primers and product densities plotted as ‘percent of input’. U251 (E & F) and U87 (G) cells (1×10^6^) were transfected with two different p300-shRNAs (Sh#1 and Sh#3) or 2 µg empty vector along with 2µg GFAP-Luc (E) or Nestin-Luc (F & G) reporter constructs. The promoter/enhancer activity was determined at 72 h posttransfection by luciferase assay. Normalized percent luciferase values are plotted as mean ± SE (n = 3). * and ** indicate p < 0.05 and p < 0.01 respectively.

We found that overexpression of p300 increased the GFAP promoter activity in a dose-dependent manner in U251 (Fig. 2A) and U87 (Fig. 2B) cells, as measured by luciferase reporter assay. On the other hand, overexpression of p300 inhibited Nestin enhancer activity in U87 cells (Fig. 2C). The inhibitory effect of p300 on Nestin enhancer activity was not measured in U251 cells because these cells had very low or undetectable basal activity.

Activation of Stat3 is implicated in the astrocytic differentiation of NSCs and GBM cells [6,15,19,54,55,56]. Based on co-immunoprecipitation of overexpressed p300 and Stat3 in 293T cells, Nakashima *et al.* have suggested that activated Stat3 binds to the interferon-γ activation site (GAS) in the GFAP promoter and recruits p300 [19]. We have previously demonstrated that GBM cells contain basal levels of constitutively activated Stat3, which can be increased in U87 cells by treatment with EGF or TGF-α and in U251 cells by treatment with IL-6 [41,49]. To examine whether p300 was recruited to the GFAP promoter in GBM cells and activated Stat3 contributed to this, chromatin immunoprecipitation (ChIP) was performed using chromatin preparations derived from IL-6-stimulated U251 and EGF-stimulated U87 cells. p300 recruitment to the GFAP promoter was detected only in U251 cells treated with IL-6 (Fig. 2D), suggesting that Stat3 activation was required for the GFAP promoter occupancy of p300 that does not directly bind to DNA [20,21]. p300 recruitment to the GFAP promoter was not detectable in EGF-treated U87 cells (Fig. 2D), which could, at least in part, be due to the differential transcriptional activity of the GFAP promoter in these two cell lines (Fig. 1F).

**Figure 3:**
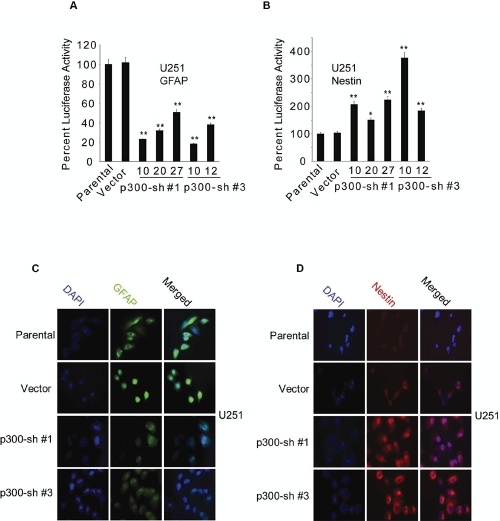
Effect of RNAi-mediated knockdown of p300 on GFAP and Nestin expression. (A & B) Five p300-shRNA stable clones (Sh#1-clones: 10, 20 and 27; Sh#3-clones: 10 and 12), one vector control and parental U251 cells (1×10^6^) were transfected with 2 µg of GFAP-Luc (A), and 2 µg of Nestin-Luc (B). Luciferase activity was measured at 72 h posttransfection and normalized percent luciferase activities were plotted as mean ± SE (n=3) (* and ** indicate p < 0.05 and p < 0.01 respectively). U251 stable clones overexpressing p300 shRNA (Sh#1-clone 10 and Sh#3-clone 10) were stained for GFAP (green) (C) and Nestin (red) (D) using specific antibodies.

Both U87 and U251 cells expressed endogenous p300 at comparable levels (Supplementary Fig. 1A), which were significantly reduced by the expression of shRNA (Supplementary Figs. 1B and C). Consistent with the observations described above, RNAi-mediated knockdown of endogenous p300 significantly reduced the GFAP promoter activity in U251 cells (Fig. 2E) while elevating the Nestin enhancer activity in U251 (Fig. 2F) and U87 cells (Fig. 2G). Two (Sh#1 and Sh#3) of the four different p300-shRNAs in pRS retroviral vector plasmid (Origene) found to be more effective than others (Supplementary Figs. 1B and C) were used in luciferase assays. To further investigate the role of p300, puromycin resistant stable clones of U251 cells expressing p300-shRNA constructs (Sh#1 and Sh#3) were generated (Supplementary Fig. 1D). These U251 stable clones had significant reduction of GFAP promoter activity (Fig. 3A) concomitant with significant upregulation of Nestin enhancer activation (Fig. 3B). A similar trend in the changes of GFAP (Fig. 3C) and Nestin protein (Fig. 3D) expression was detected by immunofluorescence. Taken together, these data show that p300 differentially regulates GFAP and Nestin expression by likely acting as an inducer of astrocytic differentiation of GBM cells.

### Inhibition of endogenous p300 enhances the invasion and migration capacities of U251 cells

**Figure 4:**
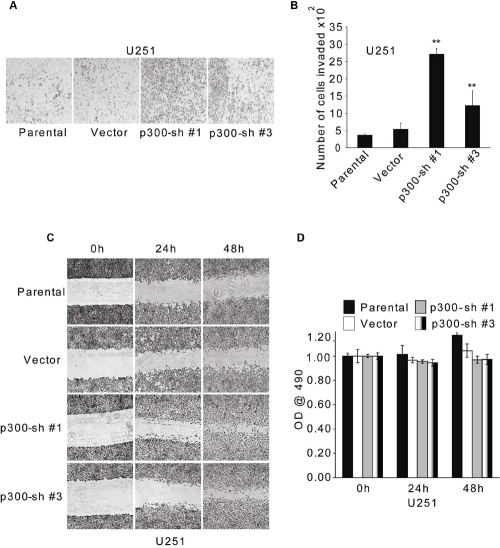
RNAi-mediated knockdown of p300 enhances invasion and migration of U251 cells *in vitro.* 2.5×10^4^ parental U251 cells, U251 stable clones expressing two different p300-shRNA constructs (Sh#1-clone 10 and Sh#3-clone 10) or empty vector were used for modified Boyden chamber invasion assay in serum-free medium. After 16 h, cells invaded to the underside of the membrane were fixed with methanol, stained with hematoxylin blue and (A) visualized using phase contrast microscopy. The number of invaded cells in (A) were counted and plotted as mean ± SE (n=3) (B). The experiment was repeated two times, and similar results were obtained. (C) U251 cells as used in (A) (parental, vector, Sh#1-clone 10, Sh#3-clone 10) were used for wound healing assay in serum-free medium and phase-contrast images were obtained at 0, 24 and 48 h after scraping. (D) Cell proliferation/viability was determined using MTT assay after 0, 24 and 48 h of serum starvation. The values are plotted as mean ± SE (n=3). ** indicates p < 0.01.

Because GSCs are implicated in the tumor invasion [13], we were interested to know whether RNAi-mediated reduction of p300 expression in U251 cells influenced their invasion capacity. Stable U251 clones expressing two p300 shRNA constructs (Sh#1 and Sh#3 in Supplementary Fig. 1D), which had significantly reduced levels of p300 exhibited a more invasive phenotype compared with both parental and vector controls in a modified Boyden chamber invasion assay [45,46,47] (Figs. 4A and B). Moreover, consistent with the invasion data, wound healing assay revealed that shRNA-mediated reduction of p300 expression substantially increased the migration capacity of U251 cells (Fig. 4C). These differences were not due to differences in cell proliferation (Fig. 4D) as determined by MTT assay [49]. Taken together, these data suggest that p300 negatively regulates the migration and invasion of GBM cells *in vitro*.

### Myc plays a role opposite to that of p300 in the transcriptional regulation of GFAP and Nestin.

**Figure 5:**
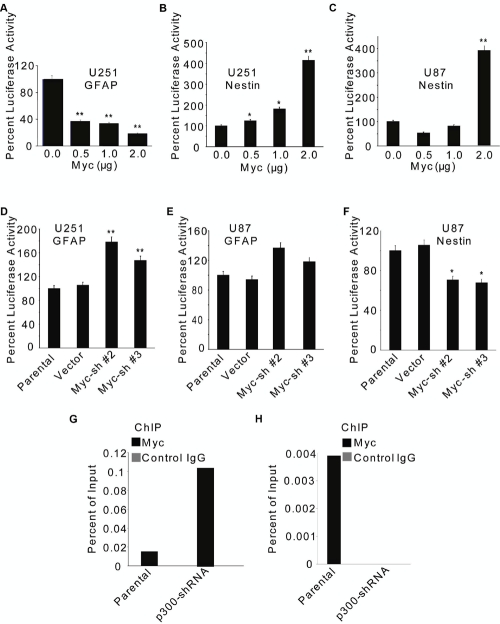
Myc differentially regulates the transcription of GFAP and Nestin genes in GBM cells. U251 (A, B & D) and U87 (C, E & F) cells (1×10^6^) were transfected with 2 µg GFAP-Luc (A, D & E) or 2 µg Nestin-Luc (B, C, & F) reporter construct along with indicated amounts of Myc expression plasmid (or empty vector) (A, B & C) or two different Myc-specific shRNAs (Sh #2 and #3) (D, E, & F). GFAP promoter (A, D & E) and Nestin enhancer (B, C & F) activities were determined at 72 h post transfection by luciferase assay and normalized percent luciferase activities are plotted as mean ± SE (n=3). * and ** indicate p < 0.05 and p < 0.01 respectively. 2×10^6^ parental U251 or p300-shRNA-expressing stable clone (Sh#1-clone 10) were treated with 20% FBS for 4 h and subjected to ChIP using anti-Myc or matched IgG antibodies (G & H). Myc recruitment to the GFAP promoter (G) or the Nestin enhancer (H) was determined by PCR using radio-labeled primers and product densities plotted as ‘percent of input’.

Recent reports suggest that Myc regulates the ‘stemness’ in GBM cells, although the underlying mechanisms remain unclear [30,31]. As with p300, we sought to investigate whether Myc regulated the GFAP promoter and Nestin enhancer activities in GBM cells. Comparable levels of Myc were expressed in U87 and U251 cells (Supplementary Fig. 2A). Overexpression of Myc resulted in reduction of the GFAP promoter activity in U251 cells (Fig. 5A) and increase in the Nestin enhancer activity in U251 (Fig. 5B) and U87 cells (Fig. 5C). Consistent with the overexpression data, knockdown of endogenous Myc by two (Sh#2 and Sh#3) of the four different shRNA constructs (in pRS vector, Origene) tested (Supplementary Figs. 3B and C) increased the GFAP promoter activity in U251 (Fig. 5D) and U87 cells (Fig. 5E). Further, RNAi-mediated knockdown Myc expression reduced the Nestin enhancer activity in U87 cells (Fig. 5F). These results suggest opposing effects for p300 and Myc on the transcription of GFAP and Nestin genes. This was further substantiated by the observations that Myc recruitment to the GFAP promoter was enhanced (Fig. 5G), and that to the Nestin enhancer was attenuated (Fig. 5H) in U251 cells that stably expressed p300-shRNA (Sh#1-clone 10), as determined by ChIP assay. Collectively, our data suggest that p300 activates the GFAP promoter activity and represses the Nestin enhancer function, whereas Myc represses the GFAP promoter activity, and antagonizes the p300-mediated repression of the Nestin enhancer function.

### Myc overrides p300 function during transcriptional regulation of GFAP and Nestin genes in GBM cells

**Figure 6:**
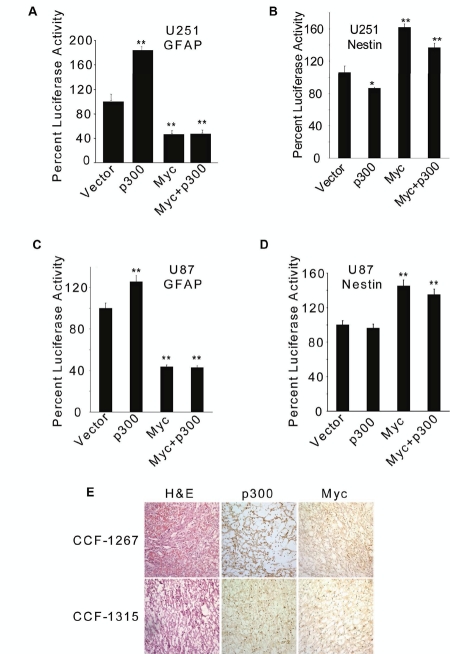
Myc overrides p300-mediated transcriptional regulation of GFAP and Nestin. U251 (A & B) and U87 (C & D) cells (1×10^6^) were transfected with 2 µg GFAP-Luc (A & C) or 2 µg Nestin-Luc (B & D) reporter construct along with 1 µg of p300 and 1 µg of Myc expression constructs, either separately or in combination. Luciferase activity was determined at 72 h posttransfection and normalized percent luciferase activities were plotted as mean ± SE (n=3) (* and ** indicate p < 0.05 and p < 0.01 respectively). (E) Sections (8 µm) of human GBM tumors (CCF-1267 and CCF-1315) were stained with p300-or Myc antibody, visualized using DAB substrate kit (Vector Laboratories) and counterstained with methyl green. Representative samples were also stained with hematoxylin and eosin (H & E).

To further understand the relative contributions of p300 and Myc to the astrocytic differentiation of GBM cells, we overexpressed both p300 and Myc in U251 and U87 cells and observed that Myc overrode the p300 functions with respect to both GFAP promoter and Nestin enhancer activities in U251 (Figs. 6A and B) and U87 cells (Figs. 6C and D). Both p300 and Myc were expressed in GBM cell lines (Supplementary Figs. 1A and 2A). To determine whether p300 and Myc were also expressed in human GBM tumors, frozen samples were sectioned (8 µm) and subjected to immuno-peroxidase staining with anti-p300 or anti-Myc antibody employing standard techniques (ABC method, Vector Laboratories, Burlingame, CA, USA), as described earlier [49]. All primary GBM tumors expressed p300 and Myc proteins, albeit at varying amounts (Supplementary Fig. 4 and Fig. 6E) Interestingly, we noted tumors, like CCF1267, that expressed relatively higher levels of Myc had cytoplasmic staining of p300 (Fig. 6E). Further, U87 cells-derived tumors grown in nude mice also expressed both p300 and Myc at varying levels (data not shown). We have used three other tumorigenic GBM cell lines, namely LN229, D54 and SNB19, and found that p300 acted as an inducer of GFAP promoter and while inhibiting the Nestin enhancer activity, as measured by luciferase reporter assay; Myc, on the other hand, repressed the GFAP promoter and activated the Nestin enhancer in these cell lines (data not shown).

In summary, our data indicate that Myc and p300 have opposing functions with regards to regulating GFAP and Nestin transcription in GBM cells. Importantly, p300 suppresses the invasion and migration potential of U251 cells *in vitro*. Moreover, the effects of Myc appear to be dominant over those of p300.

## DISCUSSION

In this article, we report three novel findings: one, p300 acts as an inducer of astrocytic differentiation of GBM cells; two, Myc overrides this p300 function and suppresses the differentiation; three, and p300 suppresses the invasive potential of GBM cells. Aside from these *in vitro* findings, we observed a reciprocal correlation between degree of astrocytic differentiation of GBM cells and their capacity of tumor formation in immune compromised rodents. It is well recognized that neoplastic cells in a tumor are heterogeneous with respect to their differentiation states and a number of recent studies suggest a hierarchical model for tumorigenesis, demonstrating that undifferentiated cancer cells, but not differentiated ones, drive cancer maintenance in rodent xenograft models [4,6,7,8,50,51]. Accordingly, our *in vivo* data support the hierarchical model for tumorigenesis in GBM model. Because each neoplastic cell of a tumor virtually represents the progeny of a single cell, a stochastic model for tumorigenesis was proposed earlier, postulating that each neoplastic cell of a tumor may retain the same potential for tumorigenesis [5]. Now, there is compelling evidence that differentiation is not an irreversible process [57,58], which suggests that these two tumorigenesis models may not be mutually exclusive.

Differentiation of stem cells, in general, is associated with their nuclear reprogramming resulting in the silencing of ‘stemness’-specific genes and induction of differentiation-specific genes [57,58,59,60]. During development of the central nervous system (CNS), differentiation of NSCs to neurons, astrocytes and oligodendrocytes is associated with major reorganization of the cytoskeleton associated with differential expression of intermediate filamentous proteins [15,17,18]. For example, Nestin and Vimentin are expressed in the early phase of CNS development and are later replaced by neurofilaments and GFAP in neurons and astrocytes respectively. In consistence with this genetic reprogramming, GSCs, like NSCs, express Nestin but not GFAP, and differentiated GBM cells do not express Nestin but GFAP [4,12,18,38,52].

Our data suggest that p300 serves as a key inducer of the astrocytic differentiation of GBM cells, which is demonstrated at the levels of GFAP- and Nestin transcription. p300 does not directly bind to DNA, but associates with chromatin through protein-protein interactions [20]. Two families of cytokines are shown to play key roles in astrocytic differentiation of NSCs: IL-6 family cytokines that activate Stat3 and BMPs that activate Smad1 [1,15,19,55]. The GFAP promoter contains a Stat3 recognition site (GAS) that recruits activated Stat3 [19]. Based on co-immunoprecipitation of overexpressed p300 and Stat3 in 293T cells, Nakashima *et al*. have suggested that activated Stat3 binds to the GAS and recruits p300 [19]. We found that expression of a mutant dominant (DN-Stat3) [34,61] resulted in an increase in Nestin enhancer activity in U87 and U251 cells (Supplementary Figs. 4A and B), while inhibiting GFAP promoter activity in U251 cells (Supplementary Fig. 4C). Further, mutation in the GAS (Fig. 1E) in the GFAP promoter dramatically reduced the luciferase activity in U251 cells (Supplementary Fig. 4D). Moreover, the recruitment of Stat3 to the GFAP promoter in both U251 and U87 cells could be demonstrated by ChIP experiments (data not shown). Thus, our data suggest that p300 could be recruited to the GFAP promoter *via* activated Stat3, which is persistent in GBM cells [49]. The enhancer element that controls the cell-specific transcription of the Nestin gene does not contain a functional GAS element; therefore, it remains to be seen how p300 is recruited to the Nestin enhancer to suppress its activity.

Because malignant glioma cells expressing neural stem cell markers exhibit a migratory potential similar to normal neural stem cells [13], we wanted to know whether p300 regulates the migration and invasion of GBM cells. Our data show that RNAi-mediated knockdown of p300 significantly increases the migration and invasion of U251 cells *in vitro*. This finding is consistent with the earlier observations by Rutka *et al.*, who have demonstrated that elimination of GFAP in U251 cells with anti-sense RNA results in the marked decrease in cell adhesion and increase in invasiveness [12], which is associated with increased expression of β1 integrin [62] and CD44, and with redistribution of actin forming actin stress fibers implicated in the regulation of cell motility [63].

Myc plays important roles in development and cancer by regulating cell cycle progression, apoptosis, transformation, differentiation and angiogenesis [22,64]. Mice overexpressing transgenic Myc under the GFAP promoter develop malignant gliomas [65]. Here, we report that Myc represses the transcription of GFAP in GBM cells with concomitant induction of Nestin, and it overrides the p300-mediated induction of GFAP and repression of Nestin. Thus, Myc suppresses the astrocytic differentiation of GBM cells. These findings are consistent with the report by Lassman *et al.* that overexpression of Myc suppresses GFAP expression and induces the expression of Nestin in mature murine astrocytes [30]. It is important to note that recent studies have identified Myc as one of the four transcription factors (Oct3/4, Sox2, Klf4 and Myc), which are capable of inducing the dedifferentiation of human and mouse fibroblasts to pluripotent stem cells [57,58]. Further, it has been speculated from these studies that Myc serves as chromatin modifier allowing Sox2 and Oct3/4 recruitment to the ‘stemness’-associated genes for the maintenance of the pluripotency of stem cells [58,66]. Therefore, it remains to be seen whether chromatin modification by Myc contributes to the ‘stemness’ of GBM cells.

## SUPPLEMENTAL FIGURE

Supplemental Figure 1

## Abbreviations used:

ChIP: chromatin immunoprecipitation
CSCs: cancer stem cells
DTT: dithiothreitol
EGF: epidermal growth factor
EDTA: ethylenediaminetetraacetic acid
GAS: gamma interferon activated sequence (site)
GBM: glioblastoma multiforme
GFAP: glial fibrillary acidic protein
GSCs: glioblastoma stem cells
IL-6: interleukin-6
MTT: 3-(4,5-Dimethylthiazol-2-yl)-2,5-diphenyltetrazolium bromide
NSCs: neural stem cells
PBS: phosphate buffer saline
RNAi: ribonucleic acid interference
TK: thymidine kinase
shRNA: short hairpin ribonucleic acid.
