# Supplementary material for: p300- and Myc-mediated regulation of glioblastoma multiforme cell differentiation
Source: Oncotarget. 2010 Aug 4;1(4):289–303. doi: 10.18632/oncotarget.139 (PMC3248108; doi:10.18632/oncotarget.139)
Supplement: Supplemental Figure 1 [file oncotarget-01-289-s001.pdf]

Supplementary Figure 1.

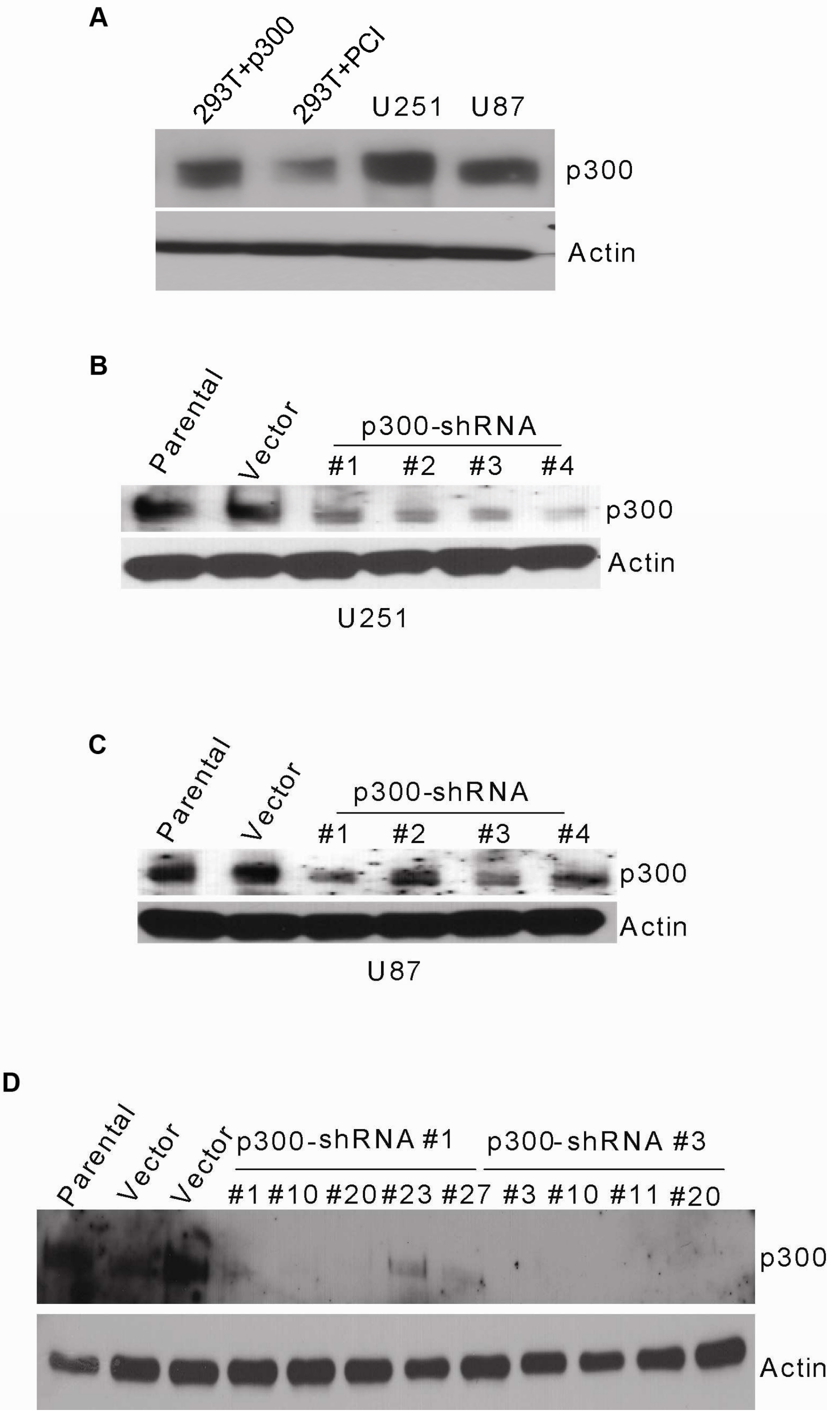

Supplementary Figure 1: RNAi-mediated knockdown of p300 in U251 and U87 cells. (A) Cell lysates derived from U251 and U87 cells, and from 293T cells transfected with p300 expression construct (as positive control) or empty vector (pCI) were subjected to immunoblotting using p300 antibody (upper panel) or βactin antibody for loading control (lower panel). U251 (B) and U87 (C) cells were transfected with four different p300-shRNA constructs or empty vector. After 48 h, cell lysates were prepared and subjected to immunoblotting using p300 antibody (upper panel) or β-actin antibody for loading control (lower panel). (D) To generate U251 clones stably expressing p300-shRNA, cells were transfected with p300-shRNA (Sh#1 and Sh#3) or empty vector and 12 stable clones were selected in the presence of 0.5 μg/ml of puromycin for 2-3 weeks, for each shRNA and vector. 5 clones of Sh#1, 4 clones of Sh#3 and 2 clones of empty vector were subjected to Western analysis using p300 antibody (upper panel) or β-actin antibody (lower panel) for loading control.

Supplementary Figure 2.

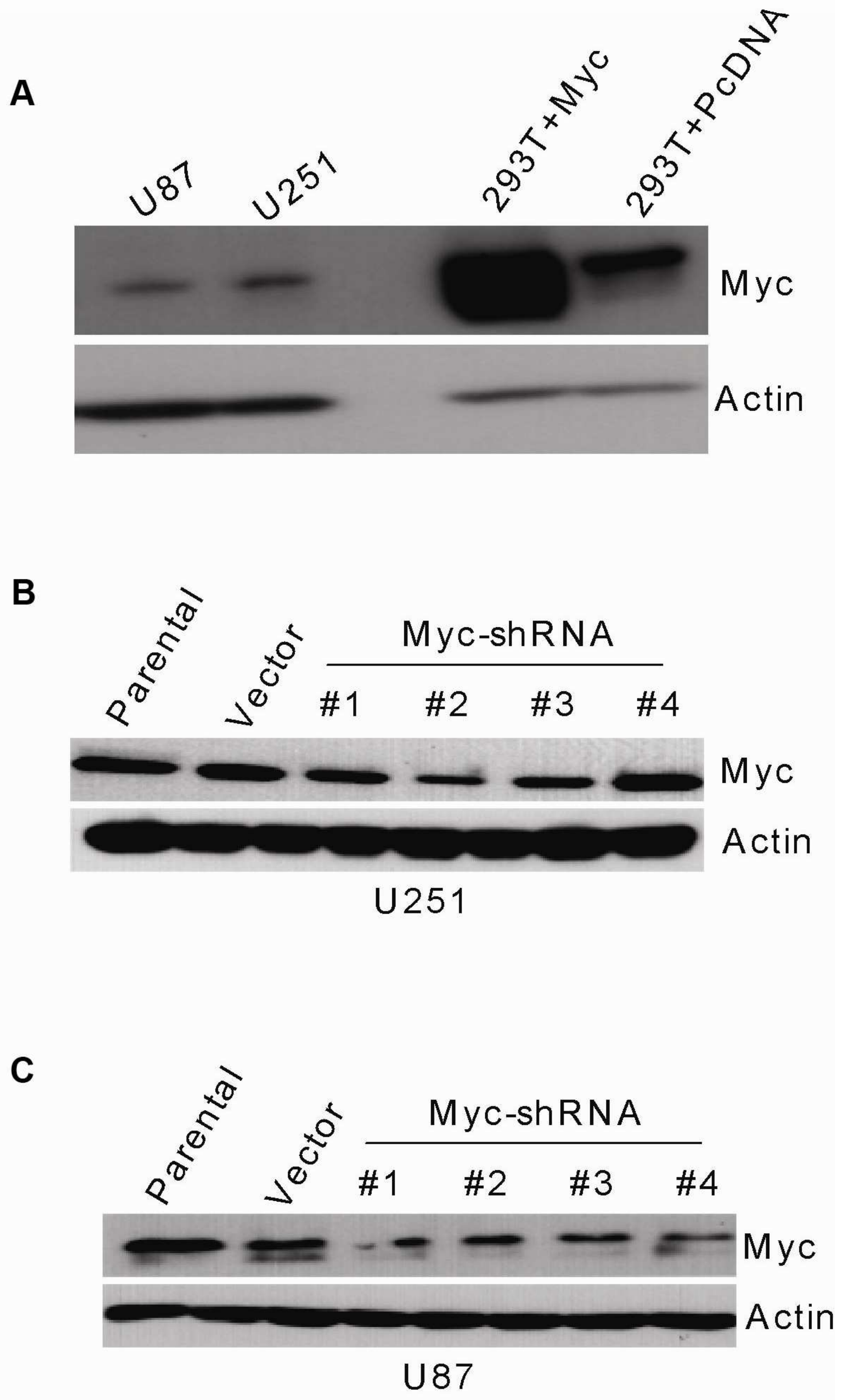

Supplementary Figure 2: RNAi--mediated knockdown of Myc in U251 and U87 cells. (A) Cell lysates derived from U251 and U87 cells, and from 293T cells transfected with Myc expression construct (as positive control) or empty vector (pcDNA 3.1+) were subjected to Western analyses using Myc antibody (upper panel), and β-actin antibody (lower panel) for loading control. U251 (B) or U87 (C) cells were transfected with four different Myc-shRNA constructs and empty vector. After 48 h, cell lysates were prepared and Myc expression was measured by Western analysis (upper panel). β-actin expression shown in the lower panel serves as loading controls.

Supplementary Figure 3.

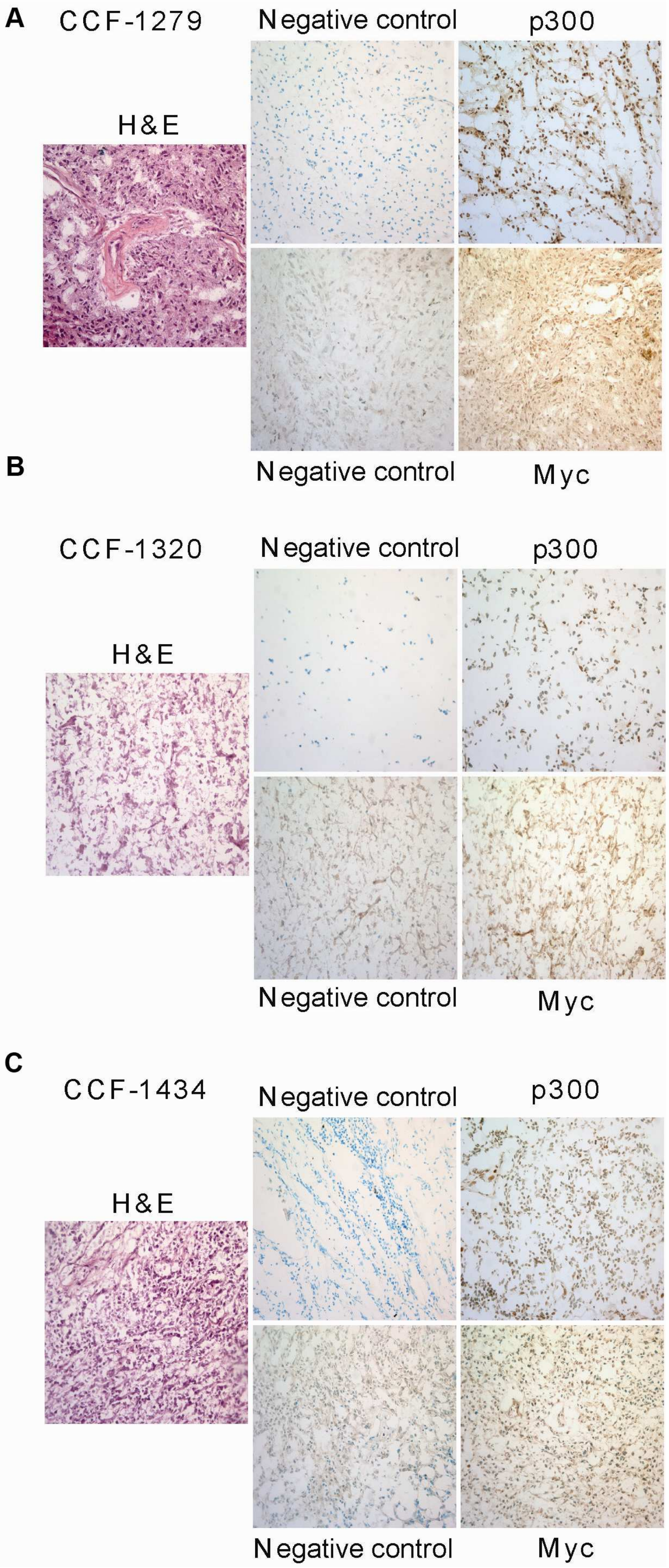

Supplementary Figure 3: Differential expression of p300 and Myc in human GBM tumors. 8 µm sections of human GBM tumors (CCF-1279, CCF-1320 and CCF1434) were stained with p300-or Myc antibody, visualized using DAB substrate kit (Vector Laboratories) and counterstained with methyl green. Samples were also stained with hematoxylin and eosin (H & E).

Supplementary Figure 4.

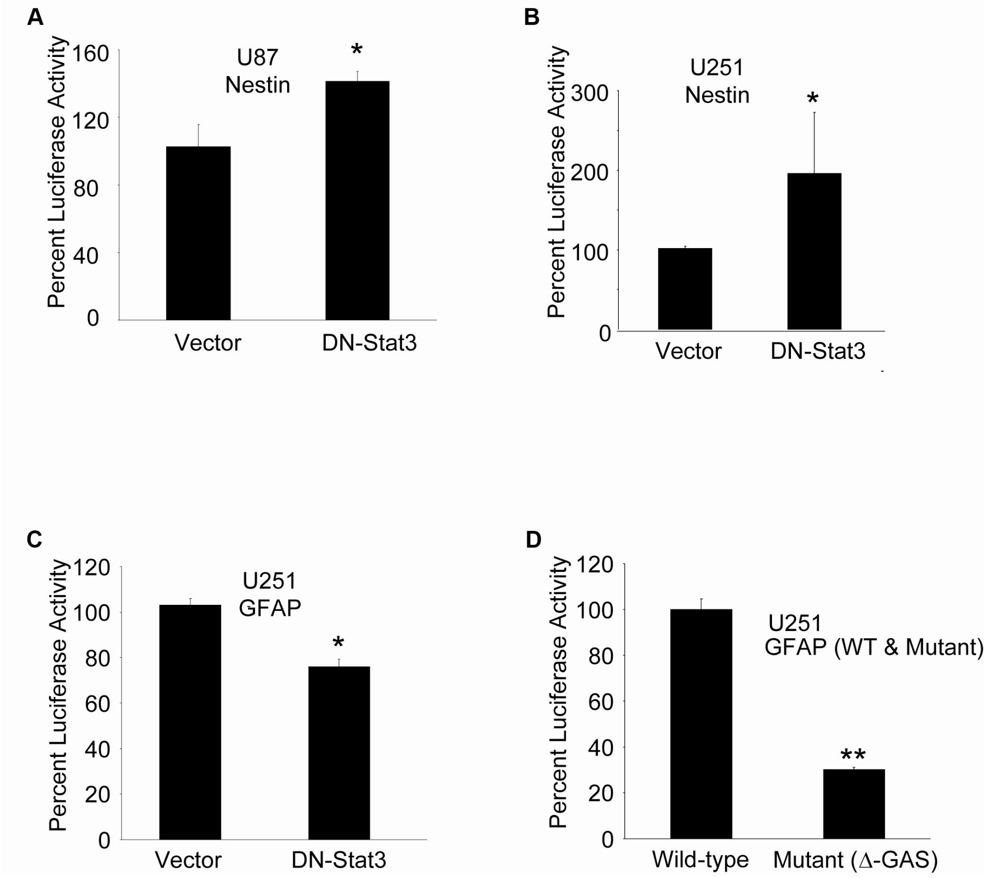

Supplementary Figure 4: Activated Stat3 regulates the transcription of GFAP and Nestin genes. U87 (A) or U251 (B & C) cells (1X10^{6}) were transfected with 2 μg luciferase reporter constructs: Nestin-Luc (A & B), and GFAP-Luc (C) along with 2 μg of DN-Stat3 construct (or empty vector). (D) 1X10^{6} U251 cells were transfected with a mutant GFAP-LUC reporter in which the GAS was mutated. The promoter/enhancer activities were determined at 72 h post transfection by luciferase assay. Normalized percent luciferase values are plotted as mean ± SE (n=3). * and ** indicate $p < 0.05$ and $p < 0.01$ respectively.
